# Public health round-up

**DOI:** 10.2471/BLT.15.010415

**Published:** 2015-04-01

**Safe listening**

A new WHO initiative targets some 1.1 billion young people worldwide, who are at risk of hearing loss due to the unsafe use of personal audio devices, including smartphones, and damaging levels of sound at noisy entertainment venues, such as shown in this photograph. The “Make listening safe” initiative was launched on International Ear Care Day on 3 March. http://www.who.int/pbd/deafness/activities/MLS

**Figure:**
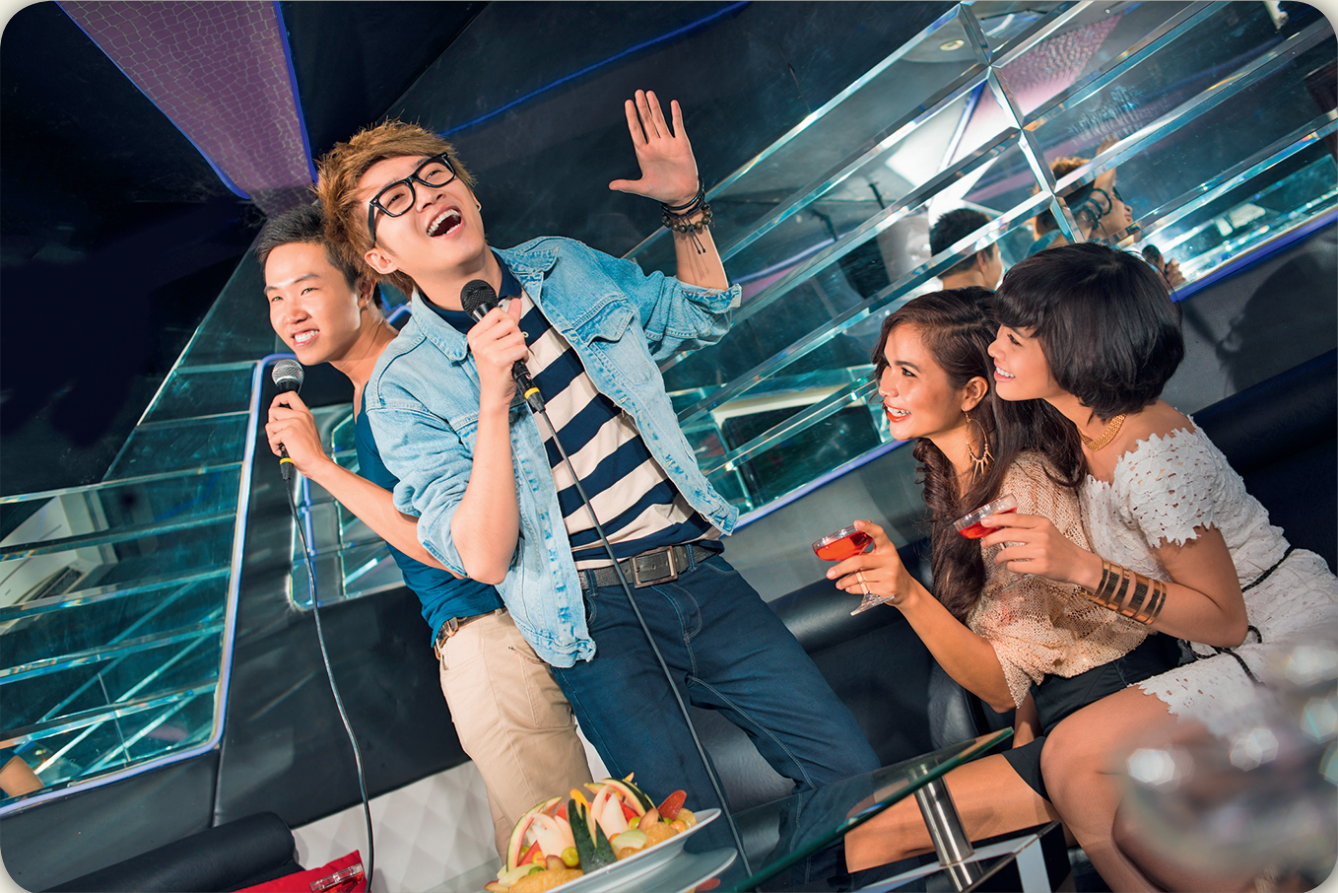
Dreamstime photos

## MERS-CoV in Saudi Arabia

Saudi Arabia has seen a surge in the number of cases of Middle East respiratory syndrome (MERS) in Saudi Arabia in recent weeks. In February, more than 50 cases were reported in several locations, including infections acquired in health facilities in Riyadh and Damman City.

A team of experts from the World Health Organization (WHO), the United Nation’s Food and Agriculture Organization, the World Organization for Animal Health and the Pasteur Institute in France visited Saudi Arabia to assess the situation and to make recommendations for improving surveillance and control, and for preventing infection with MERS coronavirus (CoV).

Members of the joint mission held discussions with representatives from the ministry of health, visited the centre that is responsible for MERS-CoV control efforts, and toured the emergency and isolation facilities of the Prince Mohammed Bin Abdulaziz Hospital.

Government officials and members of the WHO-led mission shared their concerns about the rising number of MERS-CoV cases in recent weeks and, in particular, in health-care facilities.

WHO and the Saudi Arabian health authorities identified several key areas for urgent attention. A better understanding of the modes of infection and transmission between animals and humans is needed as well as more research studies – the findings of which should be shared widely and promptly. Efforts are also needed to improve infection control, especially in health facilities, while social mobilization, community engagement and communications should be stepped up to raise more awareness about how to prevent the disease.

MERS-CoV is a viral respiratory disease caused by a coronavirus that was first identified in Saudi Arabia in 2012. MERS-CoV cases continue to occur, with sporadic cases and clusters of cases in communities and health-care settings. There is still no evidence of sustained human to human transmission.

In total since the emergence of the virus in April 2012, 1059 laboratory–confirmed cases of MERS-CoV, including at least 394 deaths have been reported to WHO, as of 11 March. More than 85% of these have been reported from Saudi Arabia.

http://www.who.int/csr/don/11-march-2015-mers-saudi-arabia

## After cyclone Pam

The WHO office for the Western Pacific Region has been coordinating response efforts with Vanuatu's Ministry of Health and other humanitarian partners to bring health support to the Pacific island country following the devastation caused by cyclone Pam.

The category five cyclone ravaged the Pacific island of Vanuatu on 13 and 14 March, with winds of more than 250 km/h and massive storm surges.

As of 16 March, there were reports of deaths and serious injuries, and that as many as 90% of homes had been severely damaged or destroyed with limited or no access to health services, food and clean water in many parts of the island.

“We are working closely with our partners to get the people of Vanuatu what they need as quickly as possible,” said Dr Shin Young-soo, WHO Regional Director for the Western Pacific. “We have activated our emergency operations centre and put a support team in place to assess needs and deploy critical resources to help in the response.”

WHO has sent in health and emergency response experts with supplies to Vanuatu to assist in the response and is working with the governments of Australia and New Zealand, as well as the United Nations Children's Fund (UNICEF) and other organizations.

http://www.wpro.who.int/mediacentre/releases/2015/20150316

## Medical supplies for Ukraine

Several tonnes of medical aid procured by WHO reached Donetsk in eastern Ukraine at the end of February, as part of a United Nations convoy. WHO's contribution consisted of medical products for HIV.

The medical kits will provide treatment for more than 38 000 people with HIV in Donetsk and Luhansk.

“Health care and health services are under immense strain in Ukraine's conflict-affected areas,” said Dr Dorit Nitzan Kaluski, WHO Representative in Ukraine.

Access to care is limited because some health facilities have been destroyed, there is a shortage of medical supplies while health professionals in the affected areas have not received their salaries and patients are unable to pay for health care themselves.

Among the estimated 5 million civilians affected by the crisis in Ukraine, those living in combat zones are particularly vulnerable, as they have limited access to humanitarian assistance. The interagency convoy is only one of the many initiatives of the United Nations and its humanitarian partners to provide relief aid to those in need.

Along with other humanitarian organizations operating in Ukraine, the United Nations Refugee Agency, UNICEF and WHO are concerned about the absence of secure humanitarian access to deliver aid to children and families affected by the conflict across the country.

Since March 2014, more than 1 million people have been displaced within Ukraine, including more than 134 000 children.

http://www.euro.who.int/en/countries/ukraine/news/news/2015/02/medical-supplies-from-who-reach-ukraine-as-part-of-united-nations-aid-convoy

**Cover photo**

Cattle forage for vegetables in India. The photograph highlights the human–animal interface from which many foodborne diseases arise; food safety is the theme of this year’s World Health Day on 7 April.

**Figure:**
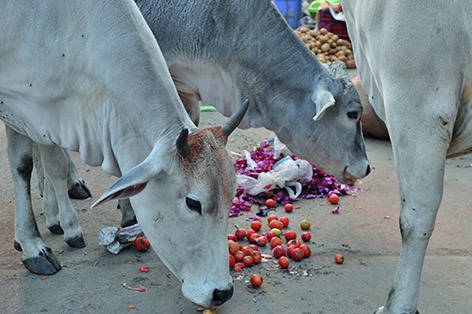
WHO/QM Islam

## Disaster risk reduction

United Nations Member States adopted a post-2015 framework for disaster risk reduction last month with a set of proposals that puts health at the heart of its goals and targets to save lives and reduce the risks of disasters.

The framework was adopted at the 3rd World Conference on Disaster Risk Reduction, held in Sendai, Japan from 14 to 18 March.

It replaces the Hyogo Framework for Action adopted at the previous World Conference for Disaster Risk Reduction 10 years ago in Kobe, Japan.

The new framework proposes different ways in which countries can strengthen their capacity for emergency and disaster risk reduction management for health and links this to the implementation of the International Health Regulations.

The framework underlines the role of science to inform evidence-based disaster risk reduction policy and practice across all sectors, and it calls for stronger partnership mechanisms to support science in the future.

The conference is the first of a series of large-scale multilateral conferences to set the post-2015 development agenda. Conferences on the Sustainable Development Goals and the United Nations’ Climate Change Conventions are due to take place later this year.

More than 5000 official participants, including heads of state, the United Nations Secretary General Ban Ki-moon, and leaders from the public health community attended, along with more than 10 000 people from the local area that was affected by the 2011 earthquake and associated tsunami and Fukushima nuclear power plant incident.

http://www.wcdrr.org/conference

## Hep B treatment guidelines

WHO issued its first treatment guidelines for chronic hepatitis B infection last month.

Several low- and middle-income countries are developing hepatitis B treatment programmes, and can use the newly-released guidance in the organization of hepatitis care and treatment services.

The guidelines fill a gap for clear evidence-based guidance in these and other low and middle-income countries and complement WHO guidance released last year on the prevention, care and treatment of infection due to the hepatitis C virus.

“Deciding who needs treatment for hepatitis B depends on a number of factors,” says Dr Stefan Wiktor, who leads WHO’s Global Hepatitis Programme. “These new guidelines give treatment recommendations that rely on simple, inexpensive tests and will help clinicians make the right decisions.”

The new guidelines recommend the use of a few simple non-invasive tests to assess the stage of liver disease to help identify who needs treatment. The guidelines provide advice on how to prioritize care for patients with cirrhosis, the most advanced stage of liver disease.

The guidelines recommend treatment for chronic hepatitis B with tenofovir or entecavir, two medicines which are available in many countries as generics, costing as little as US$ 5 per person per month.

Worldwide, an estimated 240 million people have chronic hepatitis B virus – a viral infection spread through the blood and body fluids that results in an estimated 650 000 deaths each year.

http://www.who.int/hiv/pub/hepatitis/hepatitis-b-guidelines

**Looking ahead**

**7 April – World Health Day.** This year’s theme is food safety. http://www.who.int/campaigns/world-health-day/2015/event
**18–26 May – World Health Assembly.** http://www.who.int/mediacentre/events/governance/wha
**31 May – World No Tobacco Day 2015: stop illicit trade of tobacco products. **http://www.who.int/campaigns/no-tobacco-day/2015/event

